# Esophagus‐Inspired Actuator for Solid Transportation via the Synergy of Lubrication and Contractile Deformation

**DOI:** 10.1002/advs.202102800

**Published:** 2021-10-28

**Authors:** Hui Liu, Yunlei Zhang, Shuanhong Ma, Yousif Alsaid, Xiaowei Pei, Meirong Cai, Ximin He, Feng Zhou

**Affiliations:** ^1^ State Key Laboratory of Solid Lubrication Lanzhou Institute of Chemical Physics Chinese Academy of Sciences Lanzhou 730000 China; ^2^ Center of Materials Science and Optoelectronics Engineering University of Chinese Academy of Sciences Beijing 100049 China; ^3^ Department of Material Science and Engineering University of California Los Angeles Los Angeles CA 90095 USA

**Keywords:** contractile deformation, temperature responsive systems, tubular actuators

## Abstract

Directional transportation of objects has important applications from energy transfer and intelligent robots to biomedical devices. Although breakthroughs in liquid migration on 2D surfaces or 3D tubular devices have been achieved, realizing smooth/on‐demand transportation of constrained solids within a 3D cavity environment under harsh pressurized environment still remains a daunting challenge, where strong interface friction force becomes the main obstacle restricting the movement of solids. Inspired by typical feeding mechanism in natural esophagus system which synergistically couples a lubricating mucosa surface with the peristaltic contraction deformation of the cavity, herein, this challenge is addressed by constructing an esophagus‐inspired layered tubular actuator with a slippery inner surface and responsive hydrogel matrix to realize spherical solid propulsion by photo(thermo)‐induced cavity deformation. The as‐constructed tubular actuator containing Fe_3_O_4_ nanoparticles exhibits local volumetric shrinkage upon NIR‐irradiation, which can generate large hydrodynamic pressure and considerable mechanical extrusion force (*F*_driving force_ ≈ 0.18 N) to overcome low interface friction force (*f*_friction force_ ≈ 0.03 N), enabling on‐demand transportation of constrained (pressure: 0.103 MPa) spherical solids over a long distance in an arbitrary direction. This actuator is anticipated to be used as bionic medicine transportation devices or artificial in vitro esophagus simulation systems, for example, to help formula eating‐related physiotherapy plans for patients and astronauts.

## Graphical abstract

An esophagus‐inspired layered tubular actuator with a slippery inner surface and a responsive hydrogel matrix is constructed. The as‐constructed tubular actuator containing Fe_3_O_4_ nanoparticles exhibits local volume shrinkage upon encountering remote NIR laser irradiation, which can generate large hydrodynamic pressure and considerable mechanical extrusion force, enabling on‐demand transportation of constrained spherical solids over a long distance in an arbitrary direction.

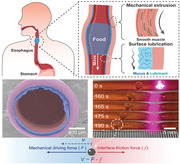

## 1 Introduction

Directional, long‐distance, and self‐propelled migration of objects on solid surfaces is crucial for many applications in bioengineering, microfluidics, and soft robotics.^[^ ^1^, ^2^, ^3^, ^4^ ^]^ It has been demonstrated that various active objects made of responsive gels, like micro‐robots, can directionally move or walk on open/unconstrained 2D planar surfaces^[^ ^5^, ^6^, ^7^, ^8^ ^]^ or within a 3D channel environment. Typically, they utilize the friction force of the gel objects against the surface, generated either by asymmetric deformation of the active material‐based objects or asymmetric (micro)structures (e.g., ratcheted) of the substrate surfaces, as the (favorable) driving force to push the objects forwards and thus enable their unidirectional movement.^[^ ^9^, ^10^, ^11^, ^12^, ^13^, ^14^ ^]^ However, in most practical mass transport applications, the objects are non‐active objects, like gas or fluid bubbles, solid food particles, drug pills/capsules, and most substances such as glass or plastic. Transporting such non‐active objects would require an active substrate able to generate peristaltic motion and thus propel the objects.

At present, the transport of liquids in specified and pre‐programmed directions has been commonly achieved on bio‐inspired 2D or 3D microstructured surfaces,^[^ ^15^, ^16^, ^17^, ^18^, ^19^, ^20^ ^]^ while the passive and active fluid transportation can be controlled in responsive polymers or characteristic structure‐integrated 3D microfluidic channel systems.^[^ ^21^, ^22^, ^23^, ^24^, ^25^ ^]^ To manipulate the liquid more intelligently within the 3D tubular environment, synthetic chemistry was used to design the channel matrix themselves with responsive features. For example, Yu et al. reported liquid crystal composite‐based tubular micro‐actuators that can remotely control transportation of viscosity‐dependent liquids via a capillary force arising from photo‐induced asymmetric deformation.^[^ ^26^ ^]^ Despite a series of breakthroughs in liquid transport, directional non‐active solid transportation in both an open and constrained environment is difficult. Meanwhile, the elastic peristaltic deformation of the channel itself under increasing internal pressure,^[^ ^27^ ^]^ becomes the key to propel the successful motion of non‐active solids. In this case, the local interface friction force acts as an unfavorable factor (rather than a driving force) that hinders the solids migration under harsh constrained or pressurized conditions (e.g., when the solid size is larger than the pipe diameter). Nistor et al.^[^ ^28^ ^]^ realized the slow transportation of solid objects within a hollow cylindrical PNIPAAM hydrogel based on the intestinal peristalsis‐like contractions mechanism, which provided new inspiration for solids transportation within tubular devices. However, the driving energy (8.3 kPa) of the suggested mode was commonly limited and only suitable for low normal pressure (≈10^2^ Pa), due to poor mechanical properties of pure PNIPAAM hydrogel and lack of highly efficient interface lubrication. Until now, intelligent/easy manipulation of solids transportation within harsh constrained/pressurized tubular environment still remains a significant challenge.

In nature, however, solid food particles of large volume (in a stuck state, suffering high constrained pressure) can smoothly pass through the esophagus into the stomach, which can be attributed to the forward driving force generated by the action of muscular peristalsis (mechanical extrusion deformation from sequential relaxation and contraction of the esophageal wall muscles), with the assistance of the slippery inner surface of the ciliated epithelium.^[^ ^29^ ^]^ This natural mechanism tells us that two key requirements of sufficient push driving force (*F*_push_, forward) and low interfacial friction force (*F_f_*, backward) must be satisfied simultaneously, in order to realize smooth transportation of solids under harsh pressurized conditions. This motivated us to develop esophagus‐inspired tubular soft actuators to push the solution of the current challenge in solid transportation. Hydrogels, a class of soft and wet materials^[^ ^30^, ^31^, ^32^ ^]^ able to deform drastically in response to external stimuli such as light,^[^ ^33^ ^]^ pH,^[^ ^34^ ^]^ temperature,^[^ ^35^ ^]^ solvent,^[^ ^36^ ^]^ magnetic field,^[^ ^37^ ^]^ and electric field,^[^ ^38^ ^]^ etc., present some promising candidate materials for deformable tubular soft actuators. Unfortunately, the current key problem is that the stimuli‐responsive volume deformation of hydrogels (PNIPAM system) is often accompanied by switching of the surface lubrication due to de‐hydration effect,^[^ ^39^ ^]^ which is unfavorable for designing esophagus‐inspired deformable devices with slippery inner surfaces to enable the solids transportation. Thus, it would be advantageous for creating a tubular actuator device if there can be a novel material design that can still retain good lubrication during a drastic mechanical deformation process under remote trigger stimulus.

In this work, we proposed to design an esophagus‐inspired tubular soft actuator based on a layered thermally‐responsive hydrogel tube with a maintained lubricated inner surface to achieve intelligent solid migration within 3D pressurized tubular environments. Such a layered tubular soft actuator was engineered by grafting hydrophilic polyelectrolyte brushes^[^ ^40^, ^41^, ^42^ ^]^ onto the inner sub‐surface of a hollow tube, which was made of initiator‐covalently‐embedded, strength/temperature‐responsive hydrogel containing photothermal Fe_3_O_4_ nanoparticles. The as‐prepared tubular soft actuator exhibited low inner surface friction force and obvious cavity shrinkage upon immersion in a high temperature water bath and upon local irradiation with a near infrared (NIR) laser. This volume shrinkage coupled with a slippery inner surface enables the tubular soft actuator to generate the high hydrodynamic pressures and large mechanical extrusion forces required to powerfully drive the directional migration of solids. With this novel biomimetic design utilizing surface chemistry and soft material mechanics, successful remote transport of solids within a 3D tubular constrained (pressure: 0.103 MPa) environment has been realized along arbitrary directions.

## 2 Results and Discussion

### 2.1 Design of Esophagus‐Inspired Soft Actuator

**Figure 1:**
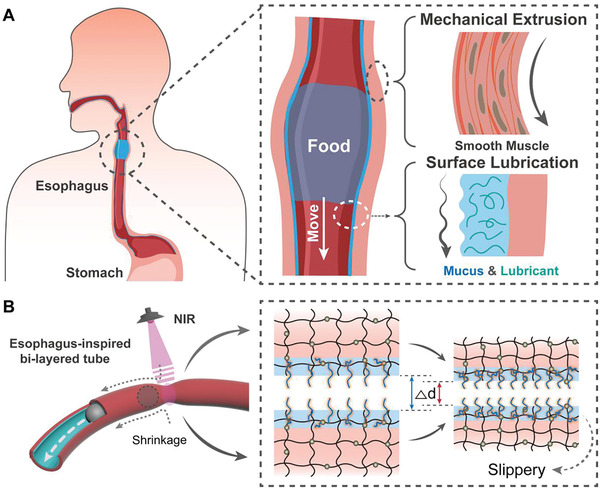
Design of esophagus‐inspired tubular soft actuator based on hydrogel tube‐g‐PSPMA (HT‐g‐PSPMA). A) The schematic diagram showing the directional passage of large‐size food from the esophagus to the stomach, based on the synergy effect of inner surface lubrication and waveform peristaltic muscle contractions. B) The schematic diagram showing the dynamic migration of a solid ball within the slippery channel of the temperature‐responsive HT‐g‐PSPMA soft actuator with Fe_3_O_4_ nanoparticles embedded, upon irradiation by an NIR laser.

As shown in **Figure** **1**A, the natural esophagus acts as a dynamic soft tube, where the mucus secreted by the mucosa provides lubrication to ease the directional transport of food from the esophagus to the stomach through the assistance of waveform peristaltic contractions of muscle.^[^ ^43^ ^]^ Inspired by this feeding mechanism, we designed and constructed a layered, temperature‐responsive tubular soft actuator with a hollow cavity and a slippery inner surface. This device exhibited volume shrinkage in response to temperature, resulting in the generation of a directional driving force to push embedded solids forward (Figure 1B).

### 2.2 Preparation and Characterization of Esophagus‐Inspired Soft Actuator

**Figure 2:**
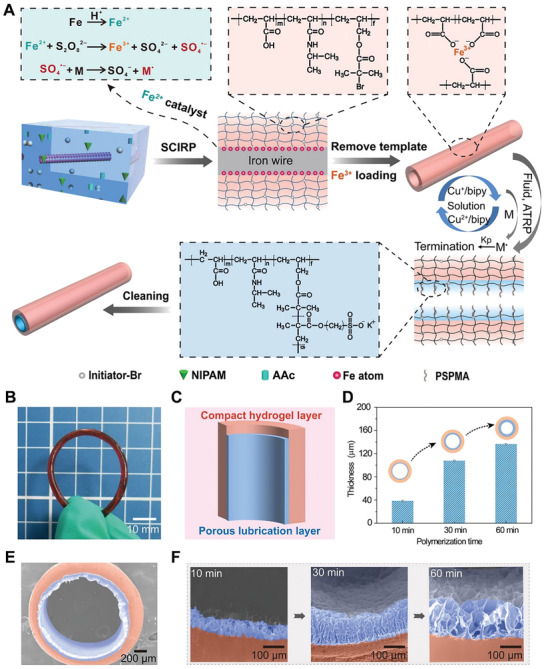
Preparation and characterizations of the HT‐g‐PSPMA soft actuator. A) The schematic diagram showing the preparation process of thermo‐responsive P(AAc/Fe‐NIPAM‐BrMA) hydrogel tube by surface catalytically initiated radical polymerization (SCIRP) method and subsequent grafting of PSPMA brushes onto inner‐surface of the tube by SI‐ATRP to generate an esophagus‐liked tubular soft actuator (HT‐g‐PSPMA). B) The photograph of the as‐prepared HT‐g‐PSPMA. C) The cross‐sectional schematic diagram of the HT‐g‐PSPMA sample. D) The thickness change of the inner lubrication layer with polymerization times of 10 min, 30 min, and 60 min. Data are presented as mean ± SD and sample size *n* = 3. E) The cross‐sectional SEM morphology of the as‐prepared HT‐g‐PSPMA sample with 30 min of polymerization time. F) The enlarged SEM images showing the interface morphology evolution of HT‐g‐PSPMA lubrication layer with increasing the polymerization time (10 min, 30 min and 60 min).

**Figure** **2**A shows the detailed preparation of the esophagus‐inspired layered soft actuator. In a typical case, the poly(acrylic acid‐isopropyl acrylamide‐2‐(2‐bromoisobutyryloxy) ethyl methacrylate) (P(AAc‐NIPAM‐BrMA)) hydrogel tubes embedded with ATRP initiator were prepared by surface catalytically initiated radical polymerization (SCIRP).^[^ ^44^ ^]^ First, the chemically crosslinked P(AAc‐NIPAM‐BrMA) hydrogel layer was formed on the surface of an iron wire when immersed into a reaction solution containing the monomer, initiator, crosslinker, and ATRP initiator. Then, the P(AAc‐NIPAM‐BrMA) hydrogel‐wrapped iron wire was immersed into an FeCl_3_ solution to generate a physically crosslinked network based on the coordination interaction between Fe^3+^ and COO^–^. The P(AAc/Fe‐NIPAM‐BrMA) hydrogel tubes with ATRP initiator were successfully prepared after removing the iron wire template. Furthermore, to provide good inner‐surface lubrication in the P(AAc/Fe‐NIPAM‐BrMA) hydrogel tube, thick poly(3‐sulfopropyl methacrylate potassium (PSPMA) brushes were grafted using the initiator‐embedded atom transfer radical polymerization (ATRP) method,^[^ ^45^ ^]^ with the assistance of a home‐made flow reactor (Figure S1, Supporting Information). As a result, a layered esophagus‐inspired slippery & responsive tubular soft actuator, defined as hydrogel tube‐g‐PSPMA (namely, HT‐g‐PSPMA), was successfully prepared (Figure 2B). The structural distinction between the slippery layer and the responsive layer was obvious (Figure 2C). The outside surface of the as‐prepared HT‐g‐PSPMA was compact (Figure S2B, Supporting Information), while its inner surface showed a porous and textured structure (Figure S2A, Supporting Information) compared to that of a pure P(AAc/Fe‐NIPAM‐BrMA) hydrogel tube (Figure S3, Supporting Information). The thickness of the inner lubrication layer increased significantly with increasing ATRP polymerization time because of the swelling‐induced embedding of polyelectrolyte brush chains into the hydrogel network (Figure 2D). The thickness increased from 38 ± 1.6 µm to 136 ± 1.4 µm when the ATRP polymerization time was tuned from 10 to 60 min. Scanning electron microscope (SEM) images were used to observe the cross‐section (Figure 2E) and the local interface (Figure 2F), confirming the layered structure. The inner surface components of the HT‐g‐PSPMA sample were further examined by X‐ray photoelectron spectroscopy (XPS) and Fourier transform infrared spectroscopy (FT‐IR). The S2p signal at 168 eV in XPS and the S=O characteristic absorption peak at 1041 cm^–1^ in FT‐IR indicate the successful grafting of the PSPMA brush (Figure S4, Supporting Information).

### 2.3 The Responsive Behavior for Esophagus‐Inspired Layered Soft Actuator

**Figure 3:**
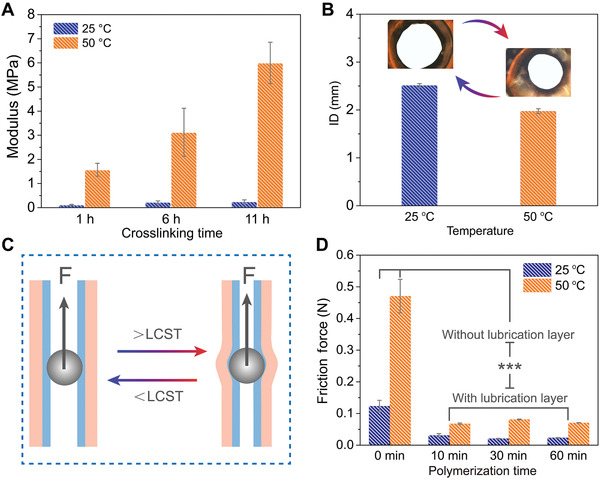
Temperature‐dependent responsive behavior investigation of the HT‐g‐PSPMA soft actuator. A) The temperature‐dependent changes of elastic modulus for the HT‐g‐PSPMA soft actuator with different physical crosslinking degree in Fe^3+^ solution at 25°C and 50°C. B) The inner diameters (ID) for HT‐g‐PSPMA sample at 25 °C and 50 °C. C) Schematic diagram showing measurements of inner‐surface friction of the HT‐g‐PSPMA against glass ball (diameter: 3 mm) upon filling the channel with 25 °C and 50°C water. D) Inner‐surface friction force of the control (without lubrication layer) and the HT‐g‐PSPMA samples (with lubrication layer) with different ATRP polymerization time at 25 °C and 50°C. *n* = 3. ***p < 0.001, compared with the without lubrication layer group both at 25 °C and 50 °C. All data are presented as mean ± SD and sample size *n* = 3.

The temperature‐dependent mechanical strength, volume shrinkage, and inner‐surface friction properties of the as‐prepared HT‐g‐PSPMA were investigated. The mechanical strengths of the tubular HT‐g‐PSPMA sample were typically temperature‐responsive and increased dramatically with increased physical crosslinking time, as the P(AAc‐NIPAM‐BrMA) hydrogel layer was immersed in the Fe^3+^ solution (**Figure** **3**A). In the typical case, the HT‐g‐PSPMA obtained with 11 h physical crosslinking exhibited a low elastic modulus (≈0.25 MPa) at 25 °C and a high elastic modulus (≈6.0 MPa) at 50 °C. Apparent volumetric shrinkages in response to temperature both for the HT‐g‐PSPMA and the blank P(AAc/Fe‐NIPAM‐BrMA) hydrogel tubes were observed by monitoring the size change of their inner diameters (ID) (Figure 3B and Figure S5, Supporting Information). The effect of the HT‐g‐PSPMA ID shrinkage on its inner‐surface lubrication was investigated. Both the HT‐g‐PSPMA and the control P(AAc/Fe‐NIPAM‐BrMA) hydrogel tubes were filled with hot or cold water respectively while solid glass balls were embedded into the channels; the friction force was obtained by moving the balls upwards while connecting them to a force sensor in a universal material test machine (Figure 3C). The friction force increased from ≈0.13 N at 25 °C to ≈0.46 at 50 °C for the control P(AAc/Fe‐NIPAM‐BrMA) hydrogel tube. For the HT‐g‐PSPMA containing the inner‐surface grafted PSPMA polymer brush lubrication layer (polymerization time: 60 min), the frictional force increased obviously from ≈0.02 N at 25 °C to ≈0.07 N at 50 °C (Figure 3D). The increasing friction of the inner‐surface in response to temperature can be attributed to the increasing mechanical extrusion force perpendicular to the apparent volume shrinkage of ID. Despite this, the friction force for the HT‐g‐PSPMA inner‐surface at high temperature was still much lower than that of the control P(AAc/Fe‐NIPAM‐BrMA) hydrogel tube.

The temperature‐responsive volumetric shrinkage of the tube allows for the control of the inner surface lubrication, directly related to the change of conformation and wettability of the polymer brush chains. The oil contact angle (OCA) of 5 µl CH_3_CH_2_Cl underwater on the inner‐surface of a control P(AAc/Fe‐NIPAM‐BrMA) hydrogel tube was measured to be 128.5° ± 1.3° at 25 °C and decreased to 114.2° ± 0.6° at 50 °C because of dehydration of the PNIPAM polymer chains (Figure S6, Supporting Information). In contrast, the OCA of the HT‐g‐PSPMA inner‐surface was measured to be 140.1° ± 4.8° at 25 °C because of sufficient surface hydration of PSPMA chains and was further increased to 145.8° ± 2.9° at 50 °C. This is attributed to the PSPMA brush chains becoming more crowded, resulting in an apparent improvement of the inner‐surface hydration and lubrication with increasing temperature. This can be confirmed by measuring the dynamic interface adhesion force between the oil droplet and the sample's surface while submerged underwater. During the dynamic contact and separation process in a 50 °C water bath, adhesion and residue of the oil droplets were observed on the inner‐surface of the control P(AAc/Fe‐NIPAM‐BrMA) hydrogel tube, while the inner surface of the HT‐g‐PSPMA sample exhibited negligible adhesion (Figure S7, Supporting Information). The inner surface adhesion force of the control P(AAc/Fe‐NIPAM‐BrMA) hydrogel tube was 44.20 ± 2.36 µN at 25 °C and increased to 48.87 ± 1.8 µN at 50 °C. In contrast, the inner surface adhesion force of the HT‐g‐PSPMA sample was 7.11 ± 0.21 µN at 25 °C and decreased to 1.48 ± 0.01 µN at 50 °C (Figure S8, Supporting Information). As a result, OCA results are in good agreement with the interface adhesion force measurement. These results indicate that the tubular HT‐g‐PSPMA sample can achieve temperature‐responsive mechanical deformation while maintaining its inner‐surface lubrication property, implying its feasible integration into an esophagus‐inspired soft actuator.

### 2.4 Migration of Solid Object within the Channel of the HT‐g‐PSPMA‐Fe_3_O_4_ Actuator

**Figure 4:**
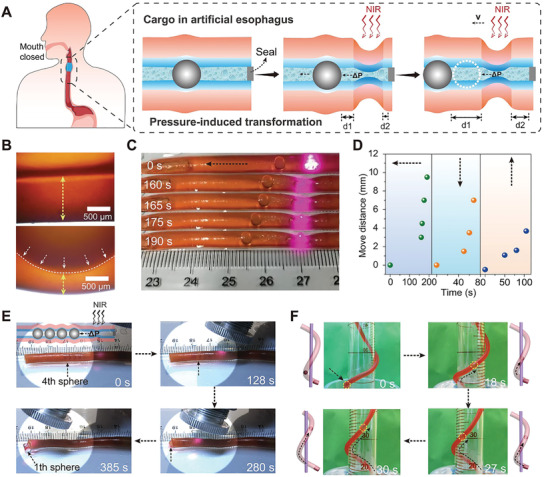
ID shrinkage‐induced hydrodynamic pressure (Δ*p*) enables the movement of solid ball. A) Schematics showing the movement of a glass ball within the channel of HT‐g‐PSPMA‐Fe_3_O_4_ actuator by ID shrinkage‐induced hydrodynamic pressure (Δ*p*) upon NIR laser irradiation (simulating the food swallowing mechanism of the human body in the mouth‐closed state). B) Cross‐sectional optical microscope images showing the (top) freeing state and (bottom) extruding & pressurizing state upon embedding a 3 mm glass ball into the channel of the HT‐g‐PSPMA‐Fe_3_O_4_ actuator. C) The photographs showing the dynamic movement of glass balls (3 mm) in the channel of the HT‐g‐PSPMA‐Fe_3_O_4_ actuator at different NIR laser irradiation times. D) Plots showing the moving distance of a glass ball (3 mm) versus irradiation time of NIR laser in the channel of the HT‐g‐PSPMA‐Fe_3_O_4_ actuator in the horizontal, vertical downwards, and vertical upwards (overcoming gravity) directions. E) The photographs showing the directional migration of four glass balls within the channel of the HT‐g‐PSPMA actuator upon NIR laser irradiation. F) The photographs showing the dynamic movement of a glass ball in a helical HT‐g‐PSPMA actuator that wraps around a cylindrical glass tube, upon gradually immersing the bottom of the tube into a 50 °C water bath.

The slippery feature of the inner‐surface for our HT‐g‐PSPMA, along with the typical thermo‐induced ID shrinkage behavior affords it the unique ability to directionally drive solids and liquids. Meanwhile, in order to realize local mechanical deformation or cavity (channel) shrinkage in response to NIR laser irradiation, Fe_3_O_4_ nanoparticles (NPs) were integrated into the HT‐g‐PSPMA. Firstly, the photothermal effects of tubular soft actuators were investigated (Figure S9, Supporting Information), the local temperature increased with extending the NIR irradiation time. In a typical case, the temperature can reach ≈51 °C after irradiating for 60 s. Importantly, the as‐prepared hydrogel tube matrix could withstand multiple cyclic deformation but without losing its NIR responsiveness, and the final outer diameter of the tube was close to that of the original tube (Figure S10, Supporting Information), implying its good reusability. As shown in **Figure** **4**A, the ID of the HT‐g‐PSPMA doped with Fe_3_O_4_ (HT‐g‐PSPMA‐Fe_3_O_4_) decreased significantly upon NIR laser irradiation to generate sufficient hydrodynamic pressure (Δ*p*) for pushing a glass ball forward upon sealing one side. This process can vividly simulate the food swallowing mechanism of the human body in the mouth‐closed state. Since the size of the glass ball (3 mm) is much larger than the ID of the tube (2.5 mm), obvious mechanical extrusion is observed (Figure 4B, down) compared to the unstressed (free) state (Figure 4B, top). The interface contact pressure can be calculated according to the reported method by Hertz contact theory.^[^ ^28^ ^]^ The average contact pressure is 0.103 MPa while the maximum contact pressure is 0.125 MPa (Section IV, supporting information). The tubular HT‐g‐PSPMA actuator can continuously drive the solid glass ball (Figure 4C, Movie S1, Supporting Information) and other kinds of balls (Figure S11, Supporting Information) within the channel to move forward in the horizontal direction, showing great generality and broad applicability of this method. Migration of glass balls is also possible in other directions, including vertically downwards (Figure S12, Movie S2, Supporting Information) and vertically upwards, overcoming gravity (Figure S13, Movie S3, Supporting Information). In a typical case, the glass ball can move 9.5 mm horizontally, 7 mm vertically downwards and 4 mm vertically upwards upon NIR irradiation of 190 s, 68 s, and 105 s, respectively (Figure 4D). The low interface friction force between the glass ball and the inner surface of the HT‐g‐PSPMA is the key factor for determining the migration distance for solid via ID shrinkage‐induced hydrodynamic pressure. By contrast, no temperature‐responsive glass ball movement was observed in the control P(AAc/Fe‐NIPAM‐BrMA‐Fe_3_O_4_) hydrogel tube channel that lacks the lubrication layer (Figure S14–S16, Movie S4–S6, Supporting Information), verifying the importance of the engineered lubrication function in such a solid transport process. Remarkably, ID shrinkage‐induced hydrodynamic pressure can even drive four glass balls to move directionally in the channel of the HT‐g‐PSPMA (Figure 4E, Movie S7, Supporting Information). Furthermore, a much larger hydrodynamic pressure was generated upon gradual submersion of a U‐shaped HT‐g‐PSPMA into 50 °C water, which enabled the transport of three glass balls within both sides (Figure S17, Supporting Information). Our HT‐g‐PSPMA actuator not only enabled the migration of a glass ball in two dimensions (2D), but also the helical movement in three dimensions (3D) overcoming gravity (Figure 4F, Movie S8, Supporting Information). Due to the existence of PSPMA polyelectrolyte brushes on the inner‐surface of HT‐g‐PSPMA, the extreme hydration and super‐hydrophilic features enabled the migration of an oil droplet without generating any channel contamination (Figure S18, Supporting Information).

**Figure 5:**
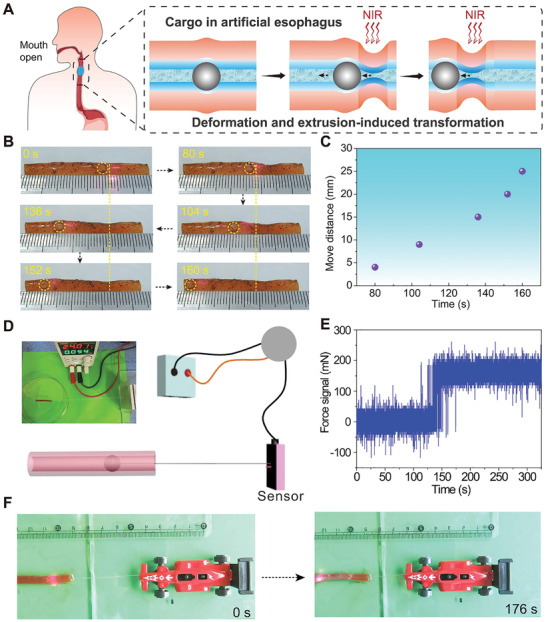
ID shrinkage‐induced extrusion deformation enables the movement of solid ball. A) Schematics showing the movement of a glass ball within the channel of the HT‐g‐PSPMA‐Fe_3_O_4_ actuator by ID shrinkage‐induced mechanical extrusion deformation upon NIR laser irradiation (simulating the food swallowing mechanism of the human body in the mouth‐open state). B) The photographs showing the dynamic movement of glass balls (3 mm) within the channel of the HT‐g‐PSPMA‐Fe_3_O_4_ actuator upon NIR laser irradiation. C) Plots showing the moving distance of a glass ball (3 mm) versus irradiation time of NIR laser within the channel of the HT‐g‐PSPMA‐Fe_3_O_4_ actuator in the horizontal direction. D, E) The photograph and schematic showing quantitative measurement of forwards driving force of a glass ball (3 mm) based on the irradiation‐induced mechanical extrusion deformation and real‐time force signal versus irradiation time recording from the sensor. F) The photographs showing the successful directional transport of a model car weighing 8 g tethered to a moving glass ball driven by NIR laser irradiation.

Apart from hydrodynamic pressure, indirect mechanical extrusion deformation generated by ID shrinkage of the HT‐g‐PSPMA can also be used to transport glass balls. The mechanism to push the glass ball involves the continuous ID shrinkage of the tube upon NIR laser irradiation to generate a forward extrusion force greater than the local interface friction force (**Figure** **5**A). This process can vividly simulate the food swallowing mechanism of the human body in the mouth‐open state. In a typical case, a glass ball (3 mm) embedded in the HT‐g‐PSPMA‐Fe_3_O_4_ could move forward continuously up to 2.7 cm within 180 s of NIR laser irradiation in the horizontal direction (Figure 5B,C, Movie S9, Supporting Information). In contrast, no apparent movement of the glass ball was observed within the channel of the control P(AAc/Fe‐NIPAM‐BrMA‐Fe_3_O_4_) hydrogel tube because of the large friction force between the glass ball and the tube wall (Figure S19, Supporting Information). Meanwhile, due to the shrinkage isotropy of the responsive hydrogel tube, the axial shrinkage effect of HT‐g‐PSPMA‐Fe_3_O_4_ on migration distance may be unavoidable. However, our layered HT‐g‐PSPMA‐Fe_3_O_4_ actuator usually shrinks locally and partly in response to NIR laser radiation when transporting objects instead of totally, which actually results in very little increase of move distance along the axial direction because of dynamic recovery (Figure S20, Supporting Information). Therefore, the contraction of the tube itself along the axial direction over a long distance is negligible. To our knowledge, this is the first demonstration of solid migration within a tubular actuator that is realized through applied mechanical extrusion deformation. A home‐made stress sensor was developed to quantitatively measure the forward driving force of a glass ball under extrusion deformation (Figure 5D). The forward driving force of a glass ball to overcome low interface friction force (*f*_friction force_ ≈ 0.03 N) could reach as high as ≈0.18 N upon applying an instant extrusion deformation by NIR laser irradiation (Figure 5E). In addition, the shrinkage/resist force of a layered HT‐g‐PSPMA‐Fe_3_O_4_ actuator during a responsive process along the axial direction was investigated, and then compared with the mechanical driving force (0.18 N: 180 mN) for transporting solids. In a typical case, the tube's two ends were fixed by stretching with ≈40 mN load (≈5% for strain), and then locally irradiated with NIR laser to test the extra strain and the force generated by this partial shrinkage. It was found that the shrinkage/resist force generated by the local contraction was only ≈20 mN (Figure S21, Supporting Information), which is far less than the mechanical driving force (180 mN), while the entire hydrogel tube did not break down. The shrinkage of the hydrogel tube in the axial direction showed little effect on successful transportation of solids; the longer tube or an anisotropic responsive hydrogel tube along the radial direction could be designed to minimize the axial shrinkage effect. Furthermore, the moving glass ball could act as a mechanical motor for directionally transporting cargo. Upon exposure to movable NIR laser irradiation, a model car weighing 8 g could move forward up to ≈2.0 cm within 180 s (Figure 5F, Movie S10, Supporting Information). These experimental results demonstrate that our tubular HT‐g‐PSPMA‐Fe_3_O_4_ actuator can mimic the natural esophageal swallowing mechanism of solids, exhibiting promising applications towards solid directional transport in the fields of micro‐actuation robots and medical devices.

## 3 Conclusions

Inspired by the typical feeding mechanism of the esophagus, through the assistance of waveform peristaltic contractions of muscle, we provide a novel conceptual route for constructing an intelligent tubular HT‐g‐PSPMA soft actuator with a lubricated inner surface and obvious thermal‐response cavity shrinkage behavior. We do so by combining the surface catalytically initiated radical polymerization (SCIRP) method and sub‐surface initiated atom transfer radical polymerization (sSI‐ATRP). The thickness of the inner‐surface lubrication layer of the tubular soft actuator could be tuned from ≈38 µm to ≈136 µm to provide a robust slippery interface that minimizes the movement resistance of a spherical solid. The cavity (channel) of the actuator exhibited temperature‐responsive shrinkage behavior to generate the necessary deformation driving force for the directional migration of solids. Moreover, an intelligent tubular HT‐g‐PSPMA‐Fe_3_O_4_ soft actuator was fabricated by integrating Fe_3_O_4_ NPs into the tube wall, exhibiting fixed‐point volume shrinkage upon NIR laser irradiation for generating large hydrodynamic pressure and considerable mechanical extrusion force (*F*_driving force_ ≈ 0.18 N) to overcome low interface friction force (*f*_friction force_ ≈ 0.03 N), realizing the on‐demand intelligent transport of a spherical solid over long distances. Intelligent solid transport was also successfully used for directionally transporting cargo. The current design can serve as a model for developing artificial in vitro therapies for patients experiencing eating difficulties, as an aid in constructing feeding simulations for astronauts, and as a platform for smarter transport devices and robots.

## Conflict of Interest

The authors declare no conflict of interest.

## Author Contributions

S. Ma and F. Zhou conceived the project idea, S. M drafted the experimental protocol, H. Liu and Y. Zhang carried out the experiments. S. Ma, X. Pei, and M. Cai supervised the work. H. Liu and S. Ma organized and wrote the manuscript. F. Zhou, X. He, and Y. Alsaid revised the manuscript. H. Liu and Y. Zhang contributed equally to this work.

## Supporting information

Supporting Information

Movie S1

Movie S2

Movie S3

Movie S4

Movie S5

Movie S6

Movie S7

Movie S8

Movie S9

Movie S10

## Data Availability

Research data are not shared.
