# Supplementary material for: Esophagus‐Inspired Actuator for Solid Transportation via the Synergy of Lubrication and Contractile Deformation
Source: Adv Sci (Weinh). 2021 Oct 28;8(24):2102800. doi: 10.1002/advs.202102800 (PMC8693057; doi:10.1002/advs.202102800)
Supplement: Supplementary file 1 — Supporting Information [file ADVS-8-2102800-s008.pdf]

WILEY-VCH

© 2021 Wiley-VCH GmbH

ADVANCED SCIENCE
Open Access

# Supporting Information

for *Adv. Sci.*, DOI: 10.1002/advs.202102800

Esophagus-inspired Intelligent Actuator for Solid-transportation via the Synergy of Lubrication and Contractile Deformation

*Hui Liu, Yunlei Zhang, Shuanhong Ma*, Yousif Alsaid, Xiaowei Pei, Meirong Cai, Ximin He*, Feng Zhou**

# Supporting Information

**Esophagus-inspired intelligent actuator for solid-transportation via the synergy of lubrication and contractile deformation**

*Hui Liu, Yunlei Zhang, Shuanhong Ma*, Yousif Alsaid, Xiaowei Pei, Meirong Cai, Ximin He*, Feng Zhou**

Ms. H. Liu, Mr. Y. Zhang, Prof. S. Ma, Prof. X. Pei, Prof. M. Cai, Prof. F. Zhou,

State Key Laboratory of Solid Lubrication, Lanzhou Institute of Chemical Physics, Chinese Academy of Sciences, Lanzhou, 730000, China.

E-mail: zhouf@licp.cas.cn; mashuanhong@licp.cas.cn.

Mr. Y. Alsaid, Prof. X. He,

Department of Material Science and Engineering, University of California Los Angeles, Los Angeles, CA, 90095 USA.

E-mail: ximinhe@ucla.edu (X.H.).

Dr. H. Liu, Dr. Y. Zhang,

Center of Materials Science and Optoelectronics Engineering, University of Chinese Academy of Sciences, Beijing, 100049, China.

# SECTION I: EXPERIMENTAL DETAILS

## 1. Materials

N-isopropylacrylamide (NIPAM, >98%), 3-sulfopropyl methacrylate potassium (SPMA, >97%) and acrylic acid (AAc, >99%) were purchased from J&K Chemicals. N,N′-methylene bis(acrylamide) (BIS), potassium persulfate (APS), 2,2'-bipyridyl (bipy, AR) andiron chloride hexahydrate ($FeCl_3{\cdot}6H_2O$) were obtained from Tianjin Chemical Reagents Corp. Initiator-Br was synthesized according to previous literature,[1] in which 2-bromoisobutyryl bromide (>98%, Energy Chemical), hydroxyethyl methacrylate (HEMA, J&K Chemical Ltd.), allyl alcohol, triethylamine and dichloromethane were used. Copper bromide (CuBr) was acquired in acetic acid by reflux. Magnetic $Fe_3O_4$ nano-particles were prepared by the method reported in literature.[2] All of the iron wires and glass balls in the experiments were acquired in China. Other general solvents were used as received.

## 2. The preparation of hydrogel tubes

The hydrogel tubes (HT) were prepared by *in-situ* SCIRP using iron wire as the template. First, the appropriate mole ratio of NIPAM and AAc monomers, initiator APS, crosslinker BIS, and initiator-BrMA were added to deionized water (40 mL) ($Fe_3O_4$ nanoparticles would be added to the above solution when preparing the hydrogel tube doped with $Fe_3O_4$) and stirred with a magnetic bar until a uniform solution was obtained. Dissolved oxygen was then removed in $N_2$ atmosphere. Next, the iron wire was immersed into above solution to carry out the SCIRP reaction, in

which the monomers were initiated by APS in the presence of BIS. The polymerization proceeded for 10 min at room temperature until a transparent hydrogel layer appeared uniformly on the wire surface. The mechanical strength of the formed hydrogel layer at that moment was observed to be relatively poor, making it difficult to directly remove from the wire to obtain an intact tubular hydrogel. To improve the mechanical strength of the hydrogel layer, the wire with the hydrogel layer was soaked into $Fe^{3+}$ solution (0.06 mol/L) for 25 min, 1, 6, 11, or 24 h. Then, the hydrogel tube was obtained after removing the iron wire. The increased mechanical strength is attributed to the physical crosslinking between the carboxyl groups and $Fe^{3+}$ ions.[3] Finally, the hollow hydrogel tube was further immersed into water to remove excess $Fe^{3+}$.

**2.1 The preparation of P(AAc/Fe-NIPAM-BrMA) hydrogel tube**

The thermo-responsive hydrogel tubes were prepared by SCIRP reaction using iron wire as the template. First, 6.78 g of NIPAM (0.06 mmol), 0.864 g of AAc (0.012 mmol), 0.02 g of APS (1% molar ratio of NIPAM and AAc), 0.01 g of BIS (0.09% molar ratio of NIPAM and AAc), and 0.153 g of initiator-BrMA (2 wt% of NIPAM and AAc) were added to the deionized water (40 mL) and stirred with a magnetic bar until a uniform solution was obtained. Then, dissolved oxygen was removed in $N_2$ atmosphere. Next, the iron wire (diameter of 1.8 mm) was immersed into the above solution to initiate the SCIRP reaction, in which the monomers were polymerized by APS in the presence of BIS. The polymerization proceeded for 10 min at room temperature. Then, after removing the iron wire, the hydrogel tube was immersed into

$Fe^{3+}$ solution (0.06 mol/L) for 24 h to enhance the mechanical strength. Finally, the hollow hydrogel tube was further immersed into water for 48 h to remove excess $Fe^{3+}$.

### 2.2 The preparation of P(AAc/Fe-NIPAM-BrMA) hydrogel tube doped with $Fe_3O_4$ (HT-g-$Fe_3O_4$)

The P(AAc/Fe-NIPAM-BrMA) hydrogel tubes doped with $Fe_3O_4$ nanoparticles were prepared by SCIRP reaction using iron wire as the template. First, 6.78 g of NIPAM (0.06 mmol), 0.864 g of AAc (0.012 mmol), 0.02 g of APS (0.12% molar ratio of NIPAM and AAc), 0.01 g of BIS (0.09% molar ratio of NIPAM and AAc), 0.153 g of initiator-BrMA (2 wt% of NIPAM and AAc), and 0.1 g of magnetic $Fe_3O_4$ nanoparticles (1.3 wt% of NIPAM and AAc) were added into deionized water (40 mL) and stirred until a uniform solution was obtained, and then dissolved oxygen was removed in $N_2$ atmosphere. Next, the iron wire (diameter of 1.8 mm) was immersed into the above solution to initiate the SRP reaction, in which the monomers were polymerized by APS in the presence of BIS. The polymerization proceeded for 10 min at room temperature. Then, after removing the iron wire, the hydrogel tube doped with $Fe_3O_4$ nanoparticles was immersed into $Fe^{3+}$ solution (0.06 mol/L) for 25 min to improve the mechanical strength. Finally, the hollow hydrogel tube was immersed in water to remove excess $Fe^{3+}$.

## 3. The preparation of P(AAc/Fe-NIPAM-BrMA) hydrogel tube-g-PSPMA (HT-g-PSPMA) and P(AAc/Fe-NIPAM-BrMA) hydrogel tube doped with $Fe_3O_4$ (HT-g-PSPMA-$Fe_3O_4$)

A circulating pump is applied for injecting ATRP monomer solution into the channel of the P(AAc/Fe-NIPAM-BrMA) hydrogel tube to perform in situ grafting of PSPMA polymer brush by sub-surface atom transfer radical polymerization (sSI-ATRP) method. The process of grafting PSPMA polymer brushes into the inside of the hydrogel tube is as follows: first, 12 mL solution of methanol/water (1:2, V/V) was obtained after stirring and bubbling with $N_2$. Then the SPMA monomer (6 g) was dissolved with continuous stirring. Next, the bipy (80 mg) and CuBr (35 mg) were adding into the above solution sequentially, in which a dark reddish-brown solution was obtained after bubbling $N_2$ gas. Furthermore, a peristaltic pump was employed to carry out polymerization inside of the hydrogel tube, where the monomer solution flows from one side of the tube at a fixed velocity (1 mL/min) for 10 min. Finally, the HT-g-PSPMA sample was acquired and repeatedly rinsed with deionized water. In addition, the polymer brushes were grafted into the hydrogel tube doped with $Fe_3O_4$ using the same method described above.

**4. Characterization**

The scanning electron microscopy images of the intended hydrogel tubes were acquired by using a JSM-5600LV scanning electron microscope (SEM, JEOL, Japan) at 20 kV. A Nicolet iS10 (Thermo Scientific, USA) Fourier transform infrared (FT-IR) spectrometer was employed to determine chemical compositions of the designed hydrogel tubes. X-ray photoelectron spectrometer (XPS) spectra were obtained to characterize the elemental composition of surface layer. The binding energy was referenced to C1s at 284.8 eV.

Mechanical tests of the resulting hydrogel tubes with different soaking time in $Fe^{3+}$ solution and different temperature were implemented using a universal material machine (EZTest, SHIMADZU) at a stretching rate of 100 mm/min. A layer of organic silicone oil was coated on the surface of the hydrogel tubes to avoid the evaporation of water before testing. The results were obtained from the mean value of at least three measurements. The equation of $u = F/A$ was employed to calculate the tensile stress, in which F is the imposed force on the hydrogel tube and A is the resulting cross-sectional area of the hydrogel tube. The cross-sectional area was acquired from $A = \pi R^2 - \pi (R\text{-}d)^2$, where d and R is the wall thickness and the outside diameter of the hydrogel tube, respectively. The modulus is defined as $E = u/e$, where $e$ is tensile strain given by the equation $e= (L - L_0)/L_0 \times 100\%$. L and $L_0$ are the real-time gauge length and the initial gauge length of the hydrogel tube, respectively.

The frictional tests of the hydrogel tubes were performed on a universal material machine by recording the force and corresponding displacement. The frictional load employed in the experiment was the glass ball that matched the diameter of the hydrogel tube. And the frictional load moved in the vertical direction of the hydrogel tube wall with a sliding rate of 50 mm/min. The distance of one sliding distance was 5 mm. Before testing, the hydrogel tube was filled with pure water, and a layer of organic silicone oil was coated on the outside to avoid the evaporation of water. The average frictional force was obtained from three consecutive measurements.

The oil adhesion force under water for the inside surface of tubular HT-g-PSPMA soft actuator was surveyed by means of a high-sensitivity microelectron

mechanical balance system (DCAT11, Data Physics, Deutschland). 5 μL of dichloroethane was installed on a metal cap suspended in water after setting the balance system to zero. Next, the oil droplet was shifted to the surface at a rate of 0.2 mm $s^{-1}$ until touching the surface. Then, the oil droplet detached from surface slowly. Finally, adhesion forces were acquired from the force-distance curves. In addition, the underwater contact angles of dichloroethane ($C_2H_4Cl_2$) were determined by using DSA-100 optical contact angle meter (Kruss Company, Ltd., Germany), in which the sample was immersed into the deionized water at low and high temperature respectively and placing an oil droplet (5 μL) on the sample surface. The above experiments were conducted three times per sample, and the average values were calculated.

To measure the mechanical extrusion force generated by shrinkage of hydrogel tube wall, a glass sphere was placed into the tube and connected to a force sensor with a thin wire. Then, the tube wrapping the glass sphere and the force sensor were firmly fixed onto a flat platform. The position of the thin wire on the sensor is adjusted to ensure that the thin wire was parallel to the platform. The hydrogel tube on the side of the glass sphere would shrink under NIR irradiation, and the glass sphere would be pushed forward. An on-demand irradiation rate was adopted to maintain quasi-static conditions. The mechanical extrusion force applied to the glass sphere was recorded by the force sensor connected via cDAQ-9171, National Instruments and processed by Labview 2017.

**5. Statistical Analysis:**

All experiments were repeated three times to verify the results, and the data were shown as mean ± standard deviation (SD). All experiments had a sample size of at least n = 3. Samples in Figure 3d were analyzed by using one-way analysis of variance (ANOVA) test. In all cases, values of $p < 0.05$ were considered as statistically significant. Statistical analysis was performed using OriginPro 2016 (OriginLab Corporation). The force signal data collected by cDAQ-9171 (National Instruments) was directly recorded by Labview 2017 without any signal processing, and these raw data was performed using OriginPro 2016 (OriginLab Corporation).

## SECTION II: MOVIES

**Movie S1:** The solid glass ball in the HT-g-PSPMA-$Fe_3O_4$ actuator is driven within the channel to move forwards in horizontal direction upon NIR laser irradiation.

**Movie S2**: The solid glass ball in the HT-g-PSPMA-$Fe_3O_4$ actuator is driven within the channel to move in vertical downward direction upon NIR laser irradiation.

**Movie S3**: The solid glass ball in the HT-g-PSPMA-$Fe_3O_4$ actuator is driven within the channel to move in vertical upwards direction upon NIR laser irradiation.

**Movies S4-S6**: The glass ball in control P(AAc/Fe-NIPAM-BrMA)-$Fe_3O_4$ hydrogel tube without lubrication layer has no apparent movements in the channel of in response to temperature in horizontal, vertical downward and vertical upwards, respectively.

**Movie S7**: Four solid glass balls in the HT-g-PSPMA-$Fe_3O_4$ actuator are driven within the channel to move in horizontal direction upon NIR laser irradiation.

**Movie S8**: The glass ball in the HT-g-PSPMA actuator is driven to helical movement in three dimensions (3D) channel in situ injecting 50 °C hot water.

**Movie S9**: The glass ball in the HT-g-PSPMA-$Fe_3O_4$ actuator move forwards continuously upon NIR laser irradiation in the horizontal direction.

**Movie S10**: The model car along with a glass ball embedded into the HT-g-PSPMA-$Fe_3O_4$ go forwards upon NIR laser irradiation in the horizontal direction.

# SECTION III: FIGURE S1-FIGURE S17

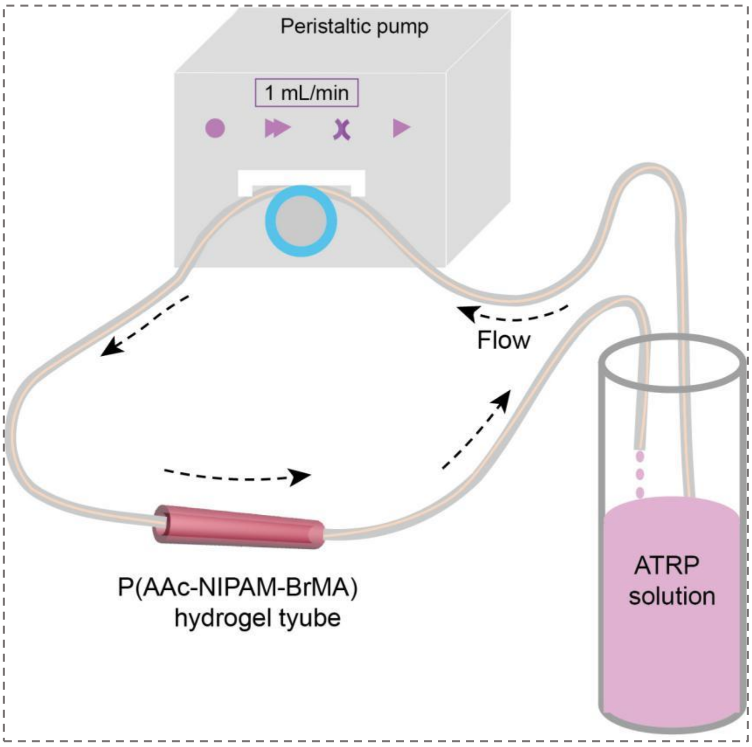


**Figure S1.** Schematic diagram showing the home-made flow reaction platform for preparing HT-g-PSPMA.

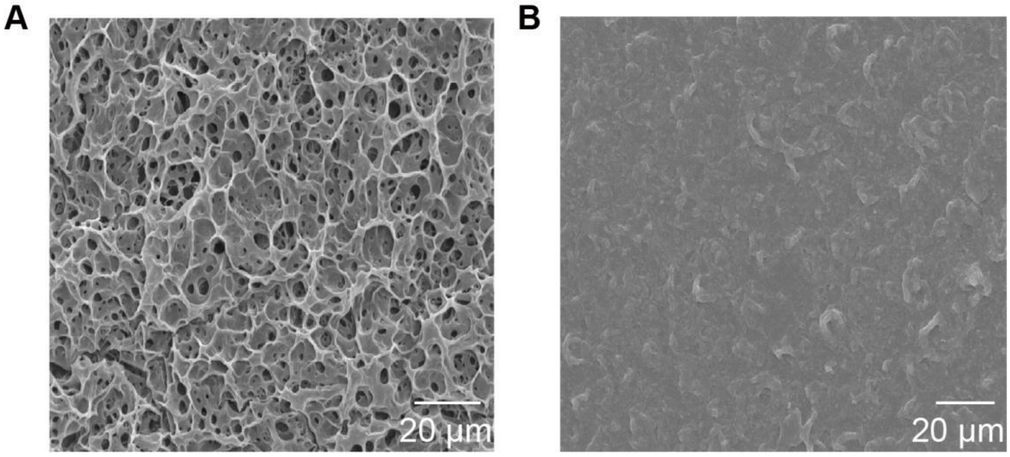


**Figure S2.** (A) The inner-surface and (B) outer-surface SEM morphologies of the HT-g-PSPMA with 60 min of polymerization time.

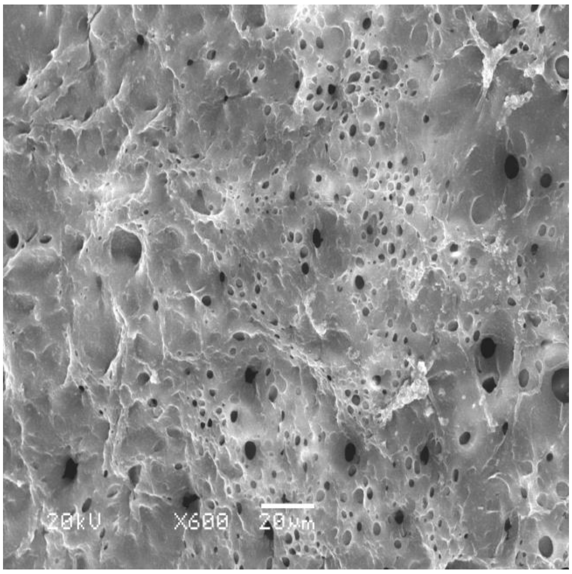


**Figure S3.** The SEM image for inner-surface of the control P(AAc- NIPAM-BrMA) hydrogel tube.

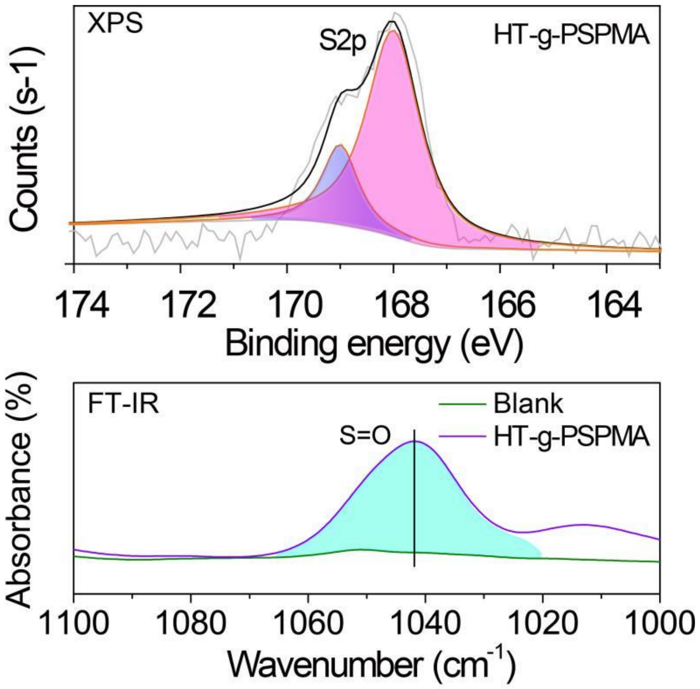


**Figure S4.** XPS and FT-IR spectrum analysis for inner-surface of the HT-g-PSPMA actuator.

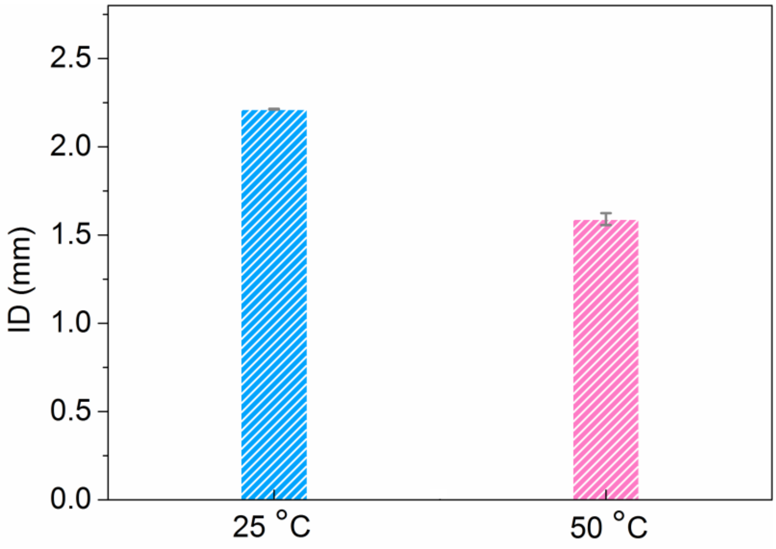


**Figure S5.** The change of inner diameter (ID) for the control P(AAc- NIPAM-BrMA) hydrogel tube in response to temperature (25 °C and 50 °C). Data are presented as mean ± SD and sample size n = 3.

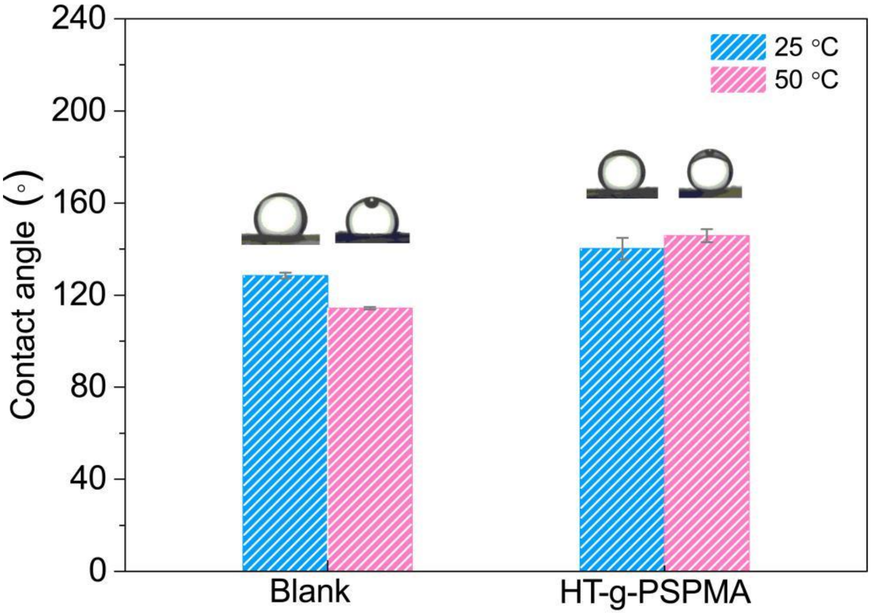


**Figure S6.** The oil contact angles (OCAs) on the inner-surface of blank P(AAc-NIPAM-BrMA) hydrogel tube and HT-g-PSPMA upon employing 5 μL $CH_3CH_2Cl$ as detection liquid droplet in response to temperature (25 °C and 50 °C). Data are presented as mean ± SD and sample size n = 3.

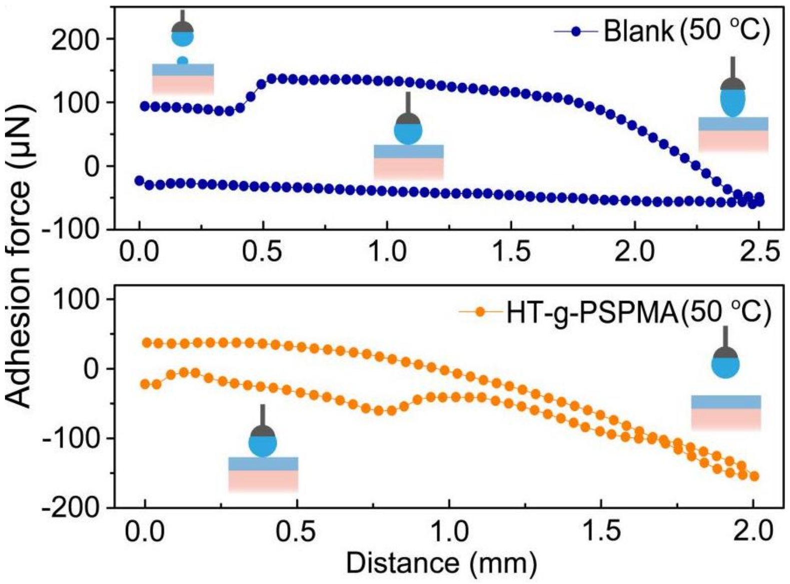


**Figure S7.** Real-time adhesion force signals vs. separation distance for inner-surface of the control P(AAc-NIPAM-BrMA) hydrogel tube and HT-g-PSPMA at 50 °C (the insert figures show the contact and separation state of oil droplet).

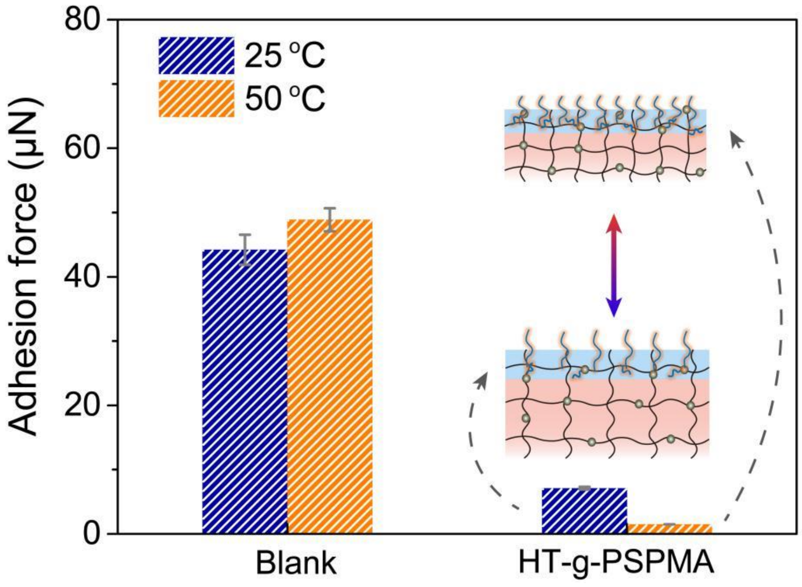


**Figure S8.** Measurement results of adhesion forces on the inner-surface of the control P(AAc-NIPAM-BrMA) hydrogel tube and HT-g-PSPMA actuator at 25 °C and 50 °C water bath (the insert figures show the  lubrication layer state of HT-g-PSPMA). Data are presented as mean ± SD and sample size n = 3.

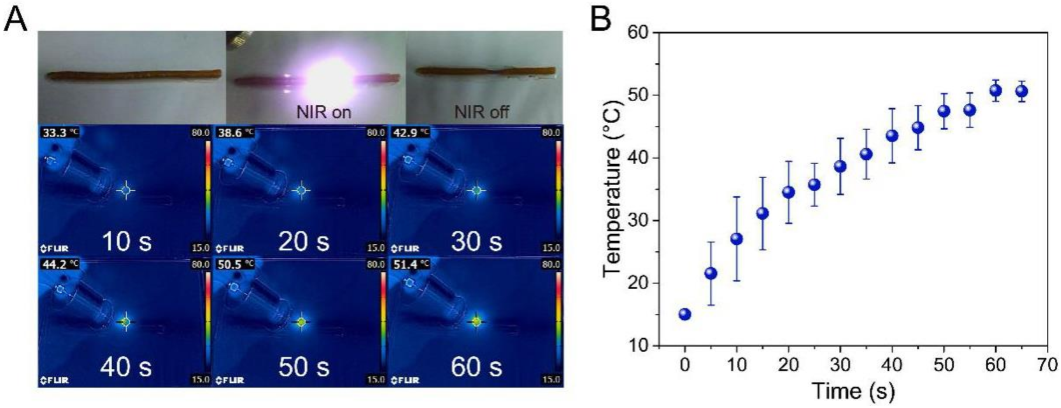


**Figure S9.** Photothermal effects of tubular soft actuators under NIR light. (A) The photos of change for tube under NIR light. (B) The curve of temperature change with the time under NIR light. Data are presented as mean ± SD and sample size n = 3.

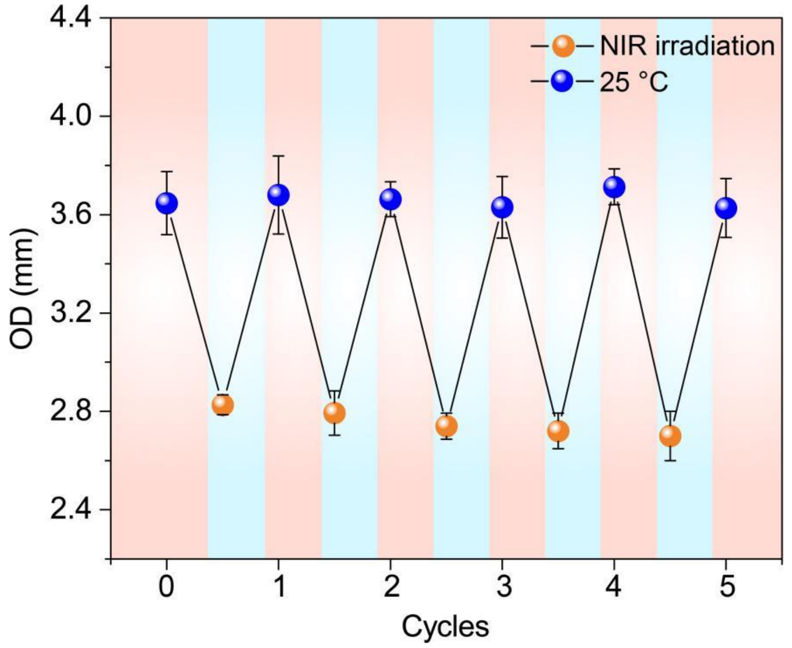


**Figure S10.** Reversible outer diameter (OD) changes for the P(AAc/Fe-NIPAM-BrMA-$Fe_3O_4$) HT-g-PSPMA-$Fe_3O_4$ by cyclically NIR irradiation and cooling at 25 °C. Data are presented as mean ± SD and sample size n = 3.

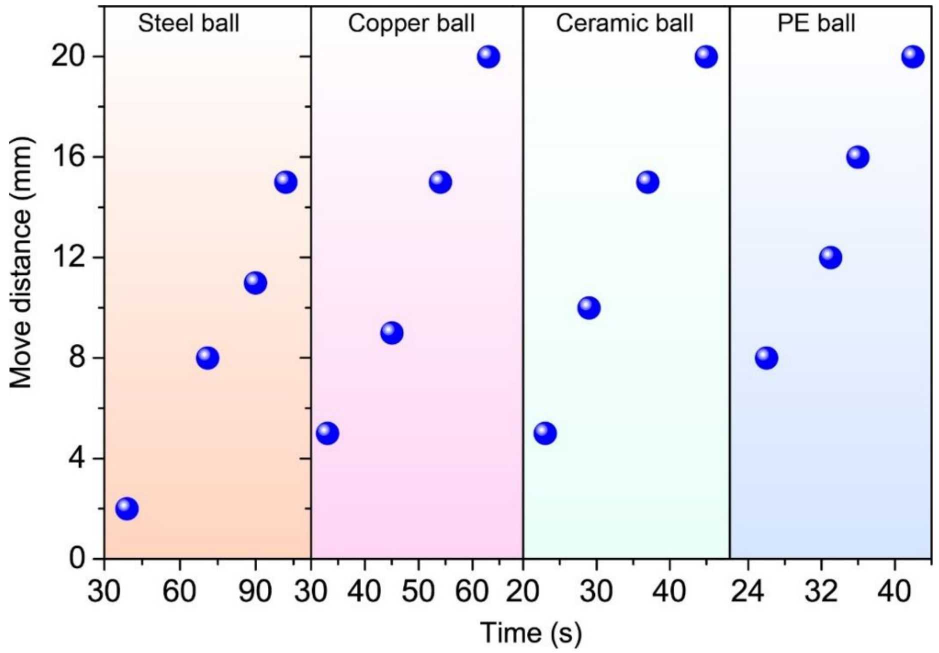


**Figure S11.** The move distance (D) of steel ball, copper ball, ceramic ball and PE ball inside the channel (inner diameter:2.5 mm) of HT-g-PSPMA-$Fe_3O_4$ upon irradiating by NIR laser in the horizontal direction. The diameters of four kinds of balls are all 3 mm.

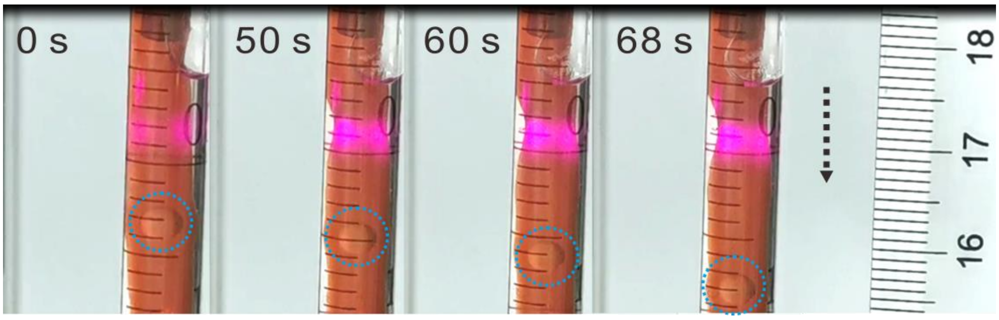


**Figure S12.** The snapshots showing the hydrodynamic pressure-induced movement trail of glass ball (3 mm) inside the channel of HT-g-PSPMA doped with $Fe_3O_4$ upon irradiating by Near infrared (NIR) laser in the downwards direction.

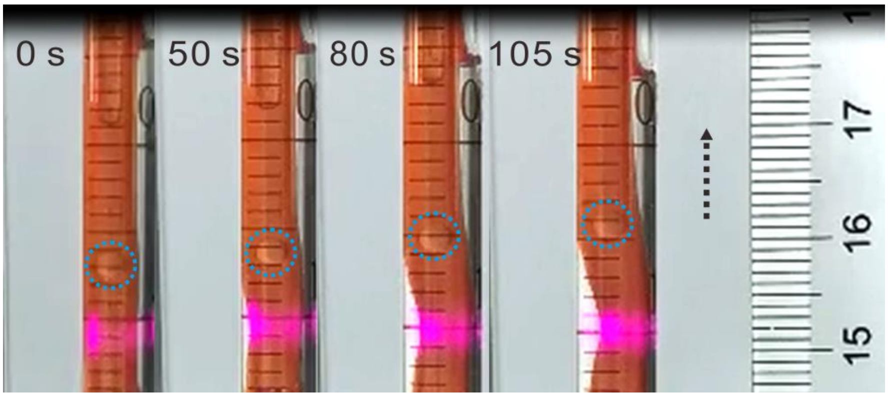


**Figure S13.** The snapshots showing the hydrodynamic pressure-induced movement trail of glass ball (3 mm) inside the channel of HT-g-PSPMA doped with $Fe_3O_4$ upon irradiating by NIR laser in the upwards direction for overcoming the gravity.

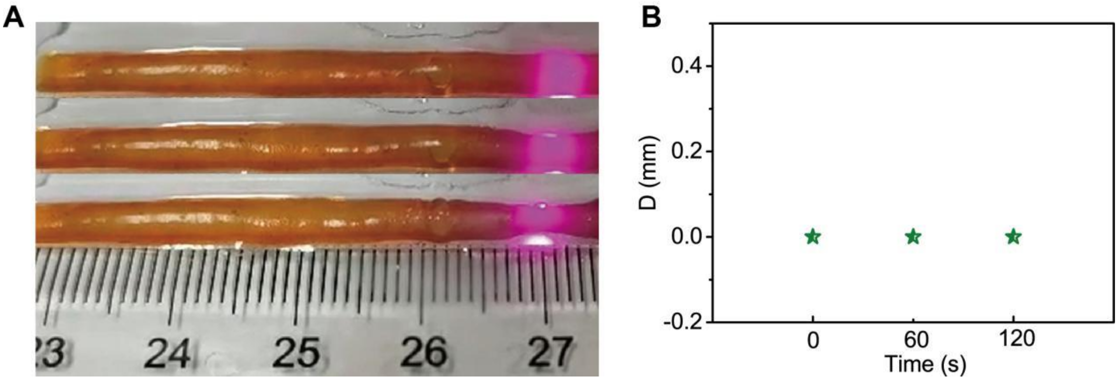


**Figure S14.** (A) The snapshots showing the hydrodynamic pressure-induced movement trail and (B) the migration distance (D) of glass ball (3 mm) inside the channel of control P(AAc-NIPAM-BrMA) hydrogel tube doped with $Fe_3O_4$ upon irradiating by NIR laser in the horizontal direction.

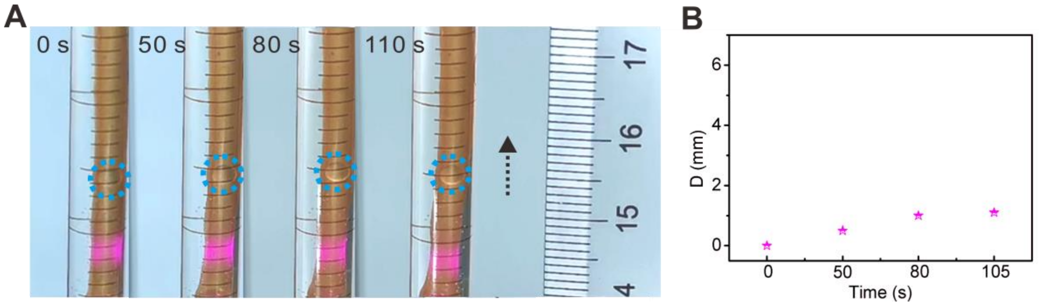


**Figure S15.** (A) The snapshots showing the hydrodynamic pressure-induced movement trail and (B) the migration distance (D) of glass ball (3 mm) inside the channel of control P(AAc-NIPAM-BrMA) hydrogel tube doped with $Fe_3O_4$ upon irradiating by NIR laser in the upwards direction for overcoming the gravity.

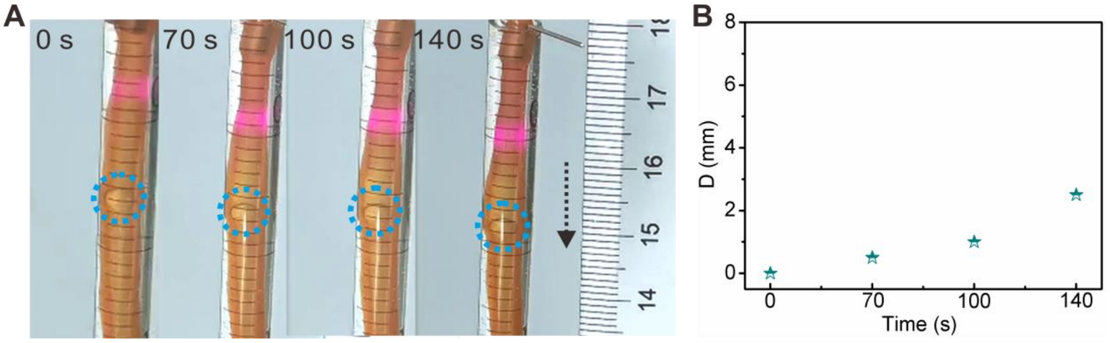


**Figure S16.** (A) The snapshots showing the hydrodynamic pressure-induced movement trail and (B) the migration distance (D) of glass ball (3 mm) inside the channel of control P(AAc-NIPAM-BrMA) hydrogel tube doped with $Fe_3O_4$ upon irradiating by NIR laser in the direction of gravity.

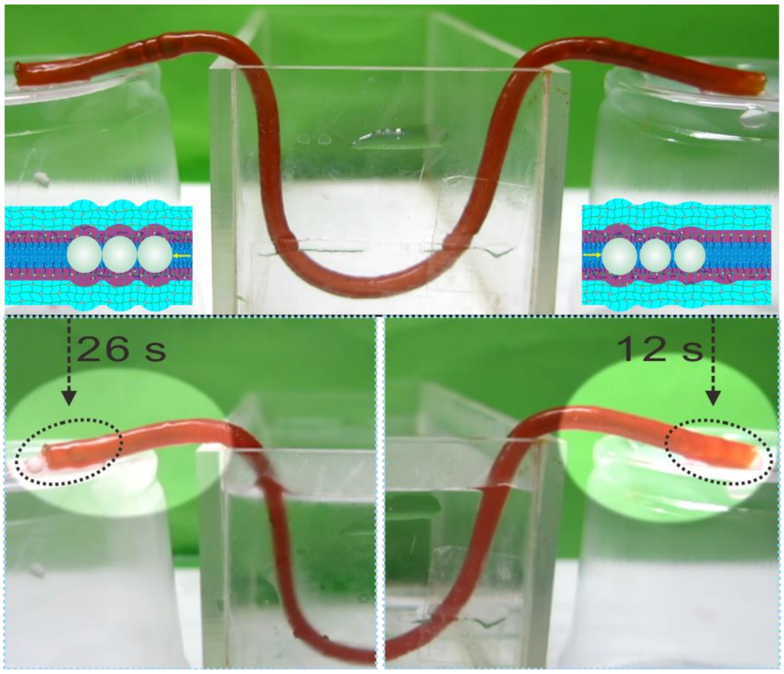


**Figure S17.** The photographs showing the dynamic movement of three glass balls within both sides of the complex HT-g-PSPMA upon gradually immersing the U-shaped part of it into 50 °C water bath.

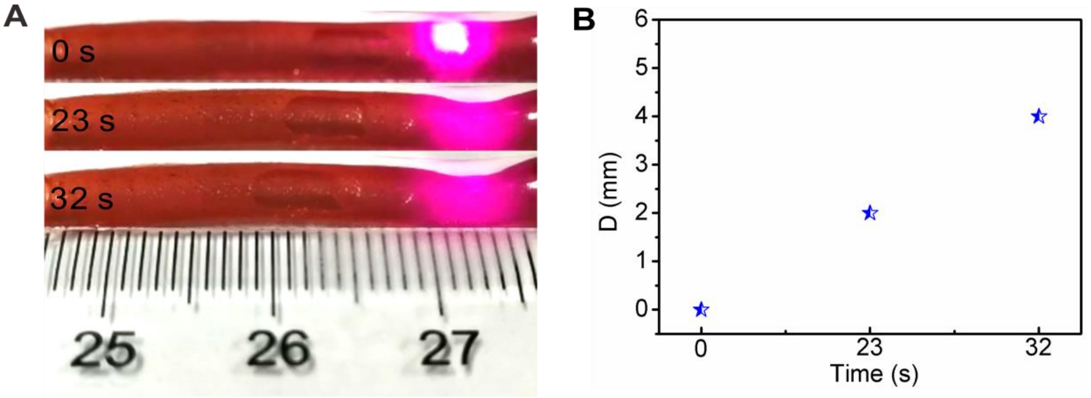


**Figure S18.** (A) The snapshots showing the hydrodynamic pressure-induced movement trail and (B) the migration distance (D) of dichloroethane oil droplets inside the channel of HT-g-PSPMA doped with $Fe_3O_4$ upon irradiating by NIR laser in the horizontal direction.

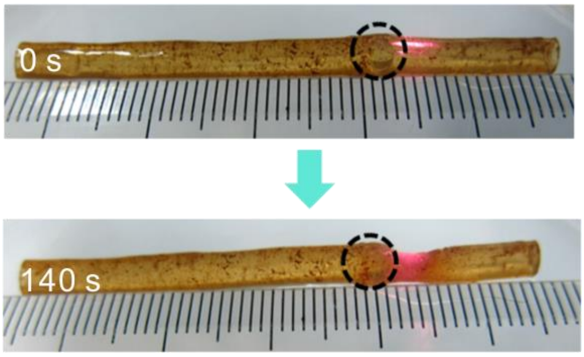


**Figure S19.** The snapshots showing the mechanical extrusion-induced movement trail of glass ball (3 mm) inside the channel of control P(AAc-NIPAM-BrMA) hydrogel tube doped with $Fe_3O_4$ upon irradiating by NIR laser in the horizontal direction (due to existing interface high friction force between glass ball and inner wall of the hydrogel tube, there is no obvious movement observed).

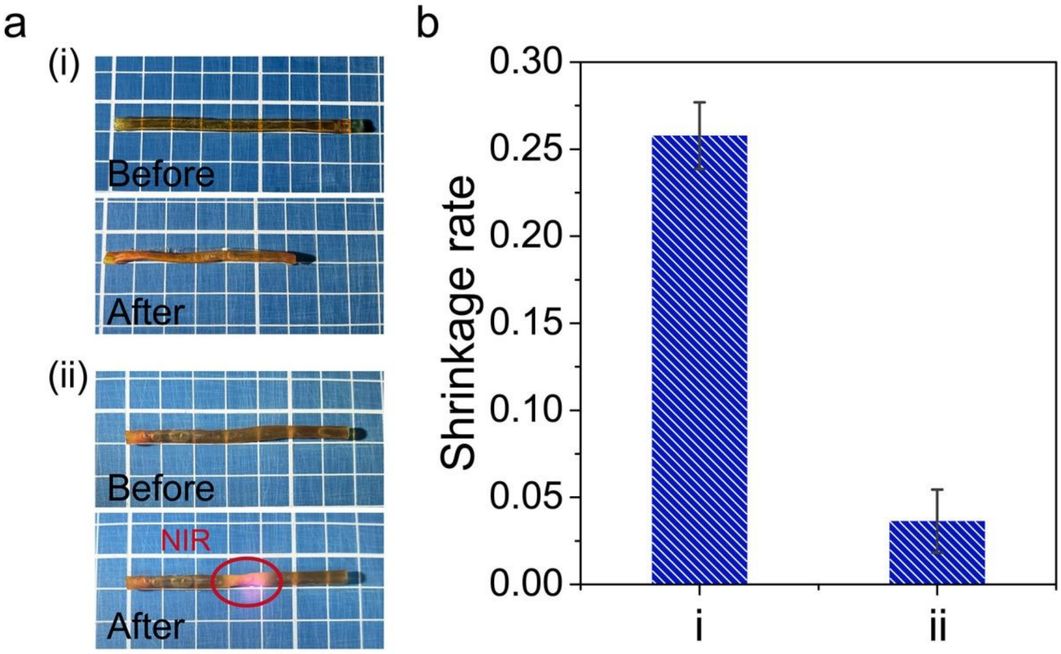


**Figure S20.** (a) The photos of HT-g-PSPMA-$Fe_3O_4$ before and after shrinking: (i) total shrinkage state of the entire HT-g-PSPMA-$Fe_3O_4$ actuator; (ii) locally and partly shrinkage state of HT-g-PSPMA-$Fe_3O_4$ actuator under NIR. (b) The shrinkage rate of the state (i) and (ii) for hydrogel tube.

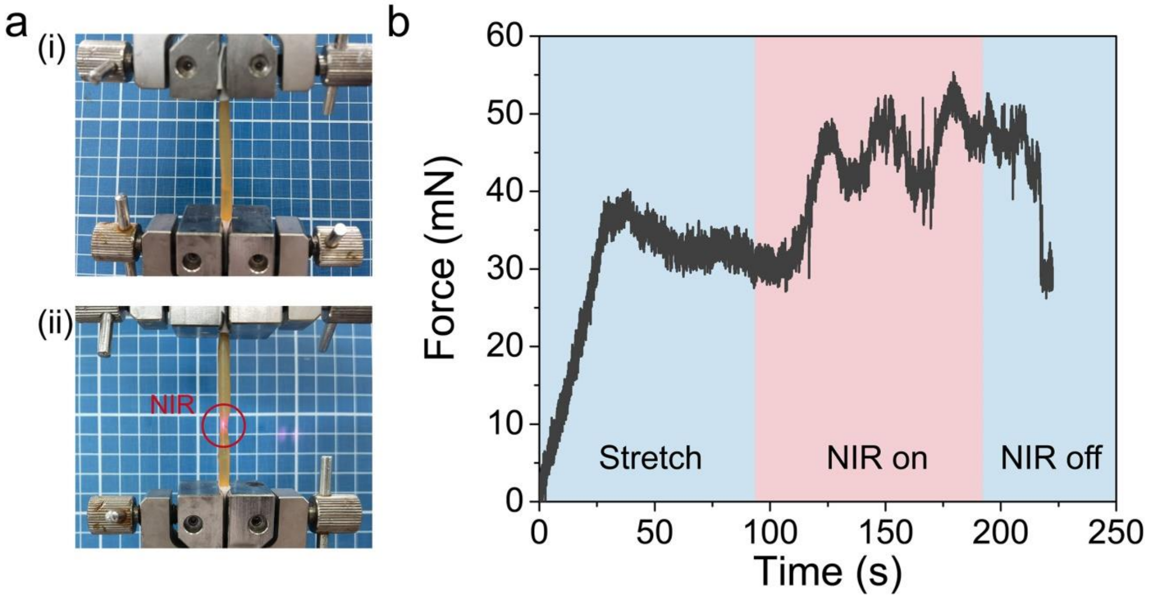


**Figure S21.** (a) Test demonstration of HT-g-PSPMA-$Fe_3O_4$ fixed at both ends and then locally irradiated by NIR. (b) The real-time force-time curve recorded in process of irradiation by NIR.

# SECTION IV: CALCULATION FOR CONTACT PRESSURE

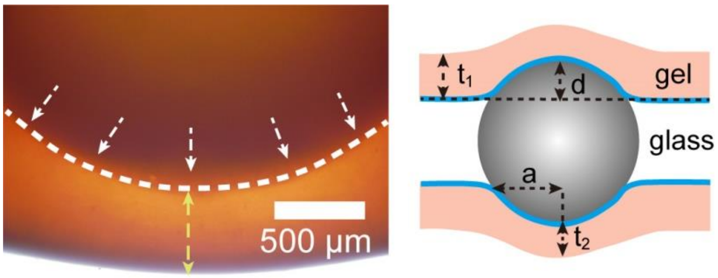


The calculation method is referred to previous method.[3] The parameters were obtained by experimental measurements. $E_{cold}$ =0.25 MPa; $R_{glass}$ =1.5 mm; $R_{gel}$ =1.25 mm; $a$~1.25 mm; $v_{gel}$=0.46; $t_1$~1 mm; $t_2$~0.5 mm.

In general, based on the Hertz contact theory, the contact half-width $a$ is a function of: (1) the reduced curvature R of interface between the spherical load and the hydrogel actuator, (2) the reduced Young's modulus E of the two materials, (3) the contact length L, and (4) the effective load W.

According to the literature, the contact radius a is: $\mathrm{a} = \sqrt{\frac{8RW}{\pi EL}}$.

The reduced curvature R is calculated from: $\frac{1}{R} = \frac{1}{R_{glass}} + \frac{1}{R_{gel}}$.

Since the glass ball is squeezed by the gel, then the collapsed section of the gel wraps around the glass ball and therefore its radius is equivalent to the radius of the glass ball, $R_{gel} = R_{glass}$, making the reduced curvature of the reduced curvature: $\mathrm{R} = \frac{1}{2}R_{glass}$. And the contact length L was $\mathrm{L} = 2\pi R_{glass}$.

The reduced Young's modulus $E$ is $\mathrm{E} = \frac{2}{\frac{1-v_{glass}{}^2}{E_{glass}}+\frac{1-v_{gel}{}^2}{E_{gel}}}$.

Since the glass ball (＞100GPa) is much stiffer than the hydrogel, $E_{glass} \gg E_{gel}$, the reduced Young's modulus can be approximated as $\mathrm{E} = \frac{2E_{gel}}{1-v_{gel}{}^2}$.

So: $E$=0.6342 MPa; $R$=0.75 mm; $W$=2.4426 N.

The contact patch between the glass ball and hydrogel tube is a spherical zone of surface: $S = 4\pi R_{glass} a$.

So, the average contact stress is $\sigma = \frac{W}{S}$, $\boldsymbol{\sigma = 0.103}$**MPa.**

The maximum contact stress is: $\sigma_{max} = E\varepsilon_{max}$, $\varepsilon_{max} = \frac{t_1 - t_2}{t_1}$, $t_1$, $t_2$ are the thickness of the hydrogel tube before and after deformation. So, $\varepsilon_{max} \sim 0.5$, $\boldsymbol{\sigma_{max} \sim 0.125 MPa.}$

# REFERENCES

[1] Von Werne, T. A., Germack, D. S., Hagberg, E. C., Sheares, V. V., Hawker, C. J., Carter, K. R. *J. Am. Chem. Soc.* **2003**, *125*, 3831.

[2] Zhu, C. H., Lu, Y., Chen, J. F., Yu, S. H. *Small*, **2014**, *10*, 2796.

[3] Lin, P., Ma, S., Wang, X., Zhou, F.. *Adv. Mater.* **2015**, *27*, 2054.

[4] V. Nistor, J. Cannell, J. Gregory, L. Yeghiazarian, *Soft Matter* **2016**, 12, 3582.
